# Ventricular tachycardia – an atypical initial presentation of sarcoidosis: a case report

**DOI:** 10.1186/1752-1947-7-196

**Published:** 2013-07-26

**Authors:** Meera Ekka, Sanjeev Sinha, Raghunandan Purushothaman, Nitish Naik, Rajiv Narang, Lavleen Singh

**Affiliations:** 1 Department of Emergency Medicine, All India Institute of Medical Sciences, New Delhi, India; 2 Department of Medicine, All India Institute of Medical Sciences, New Delhi, India; 3 Department of Cardiology, All India Institute of Medical Sciences, New Delhi, India; 4 Department of Pathology, All India Institute of Medical Sciences, New Delhi, India

**Keywords:** Cardiac sarcoidosis, Sarcoidosis, Ventricular arrhythmias

## Abstract

**Introduction:**

Symptomatic cardiac involvement is seen in less than 5% of all cases of sarcoidosis. Although clinically apparent cardiac sarcoidosis is an uncommon entity, ventricular tachyarrhythmias as the first presenting symptom are very rare.

**Case presentation:**

We discuss the case of a 41-year-old Asian woman who presented to our hospital with intermittent palpitation and on evaluation was diagnosed to have systemic sarcoidosis with cardiac involvement. She was started on multiple antiarrhythmic drugs and corticosteroids without any satisfactory response.

**Conclusions:**

Our case report indicates that sarcoidosis can manifest as ventricular tachycardia without any detectable systemic findings. This makes sarcoidosis an important diagnostic consideration in patients with ventricular tachycardia of unknown origin given the high mortality associated with ventricular tachyarrhythmias.

## Introduction

Sarcoidosis is a multisystem granulomatous disorder of unknown etiology that mainly affects the lungs, skin, eyes, and lymphoreticular system, with cardiac involvement being a rare entity. A recent review of sarcoidosis placed cardiac involvement at 2%; it is one of the least common manifestations [1]. However, cardiac involvement may be an asymptomatic accompaniment to pulmonary disease or may be the presenting features of systemic sarcoidosis. Cardiac involvement with sarcoidosis is found at autopsy in approximately 25% of patients with the disease; however, only 2 to 5% of all patients with sarcoidosis have clinically significant cardiac symptoms such as congestive heart failure, heart block, ventricular arrhythmia, or sudden death [2].

Although the disease can manifest itself with comparatively benign signs and symptoms, cardiac involvement can prove to be fatal [3]. The early identification and prompt treatment of cardiac sarcoidosis can reduce the chances of sudden cardiac death [4]. Here we present the rare case of a patient who had no history of systemic sarcoidosis before she presented with refractory monomorphic ventricular tachycardia (VT) with a fatal outcome. In our case report we would like to draw attention to the importance of ruling out cardiac involvement in any cases with systemic sarcoidosis to prevent unfavorable outcomes.

## Case presentation

A 41-year-old Indian woman presented with a history of intermittent palpitations of 10 days’ duration to our hospital. She denied any history of fever, cough, chest pain, dyspnea or syncope or relevant past history of these. She had a past history of pulmonary tuberculosis in 1992 with 6 months of antitubercular treatment. She denied any past history of hypertension, coronary artery disease, diabetes mellitus, thyroid or connective tissue diseases. On examination she was afebrile with a regular pulse of 142 beats/minute, and a blood pressure of 120/70mmHg. She had brownish, waxy papules in the skin of her bilateral lower limbs above her medial malleolus but the rest of the general physical examination did not reveal any abnormal findings. Her systemic examination was within normal limits. She was transferred from our emergency department to the cardiac coronary unit and a synchronized electrical cardioversion successfully terminated her VT.

**Figure 1:**
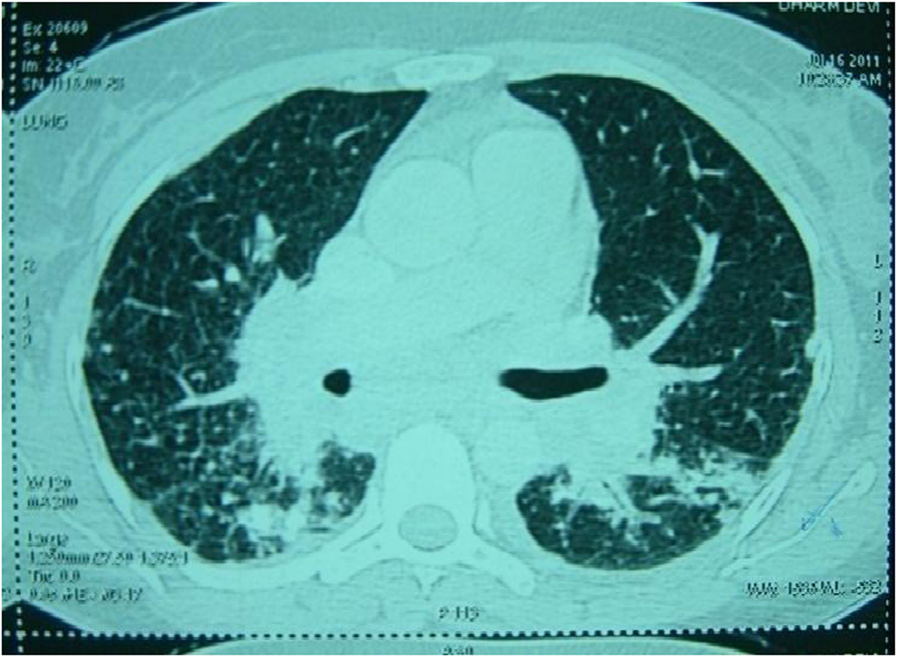
High-resolution computed tomography scan of the chest showing peribronchial and subpleural nodules, interstitial thickening and bilateral hilar lymphadenopathy.

**Figure 2:**
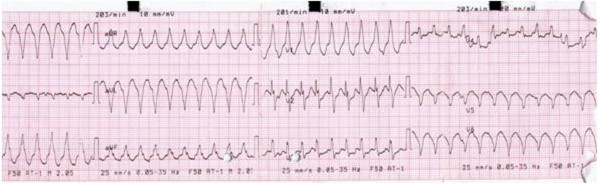
Electrocardiogram showing monomorphic ventricular tachycardia.

On investigation, her hemogram, blood biochemistry, liver, renal function and thyroid function were within normal limits. Her angiotensin-converting enzyme level was 147μL (normal range: 0 to 20). Her serum calcium was normal. Her immunological markers were negative with normal erythrocyte sedimentation rate and C-reactive protein. A contrast-enhanced computed tomography (CT) chest scan showed findings suggestive of stage-III sarcoidosis (Figure 1). A slit lamp examination of her eyes did not reveal any abnormal findings. An electrocardiogram showed monomorphic VT and right bundle branch block with right axis deviation (Figure 2).

**Figure 3:**
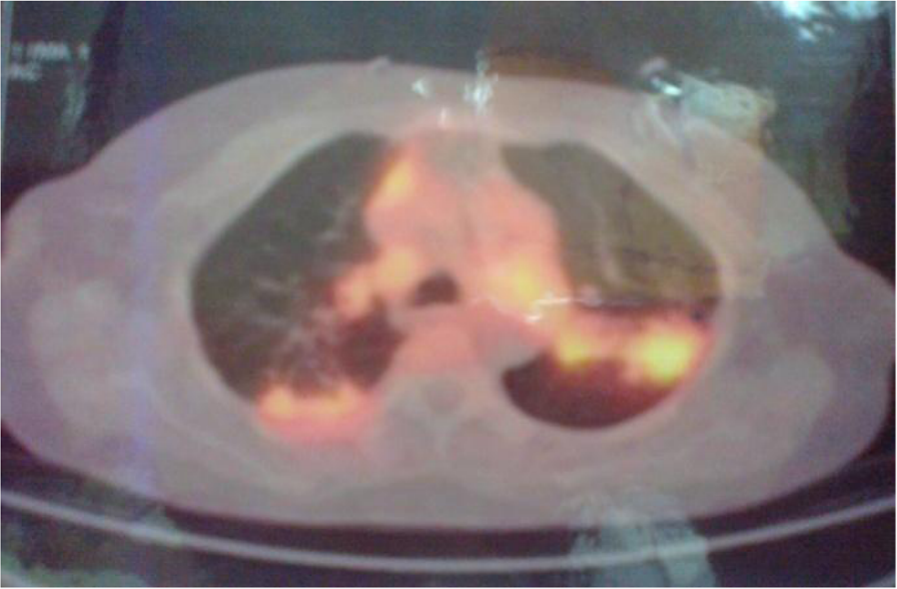
**Evidence of active disease in bilateral lungs and mediastinum shown on **^**18**^**F-fluorodeoxyglucose positron emission tomography scans.**

**Figure 4:**
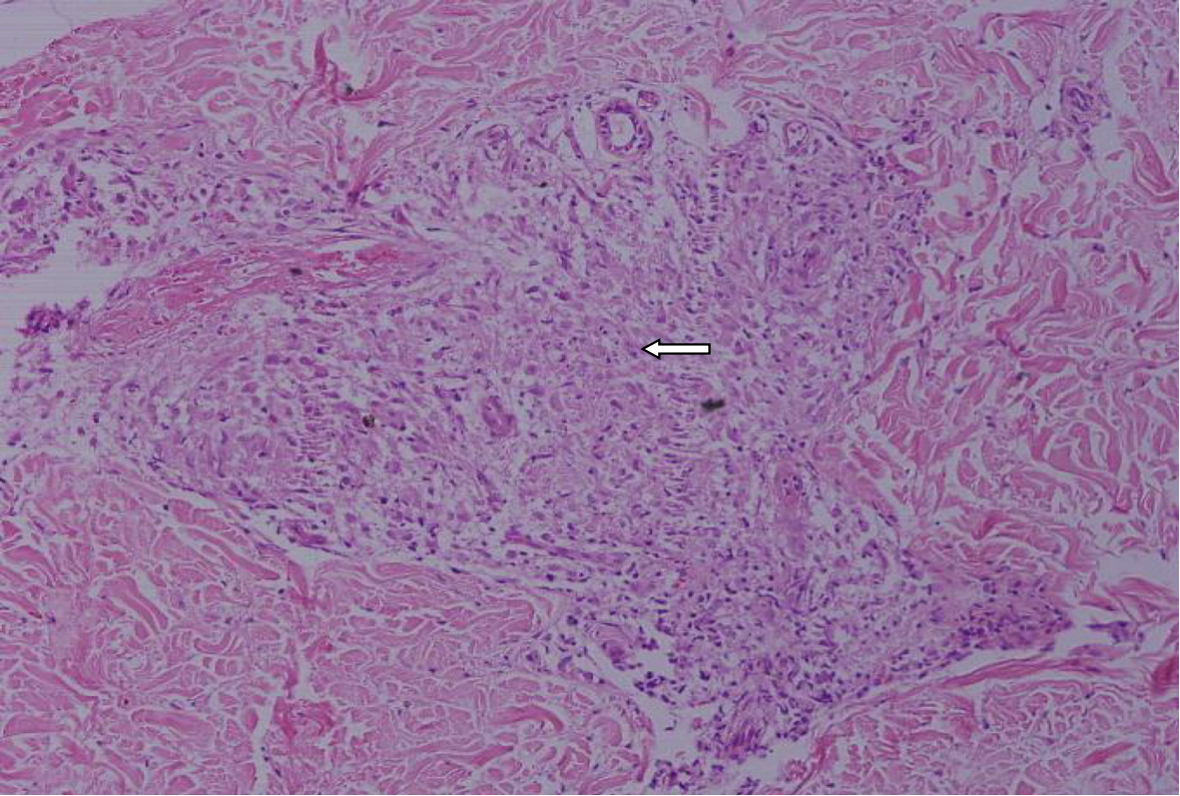
Skin biopsy showing non-caseating discrete granuloma (arrow) (×200, hematoxylin and eosin stain).

To evaluate the extent of the disease an ^18^F-fluorodeoxyglucose positron emission tomography-computed tomography (PET)-CT scan was performed. The study showed evidence of active disease involving her bilateral lungs, mediastinal, right supraclavicular, peripancreatic and retrocrural lymph nodes with focal lesions in segments VII and VIII of her liver (Figure 3). As the supraclavicular node was not palpable, a biopsy was attempted from the superficial lesion in the right lobe of her liver. This biopsy showed interstitial fibrosis but no evidence of epithelioid granulomas. A skin biopsy from a waxy papule of her lower limb showed non-caseating epithelioid cell granuloma suspicious for sarcoidosis (Figure 4). A Ziehl–Neelsen stain showed an absence of acid-fast bacilli. A diagnosis of systemic sarcoidosis with suspected cardiac involvement was made. Bronchoscopy with transbronchial lung biopsy was not attempted because systemic sarcoidosis had already been confirmed with skin biopsies and further intervention in an unstable patient was considered unnecessary.

**Figure 5:**
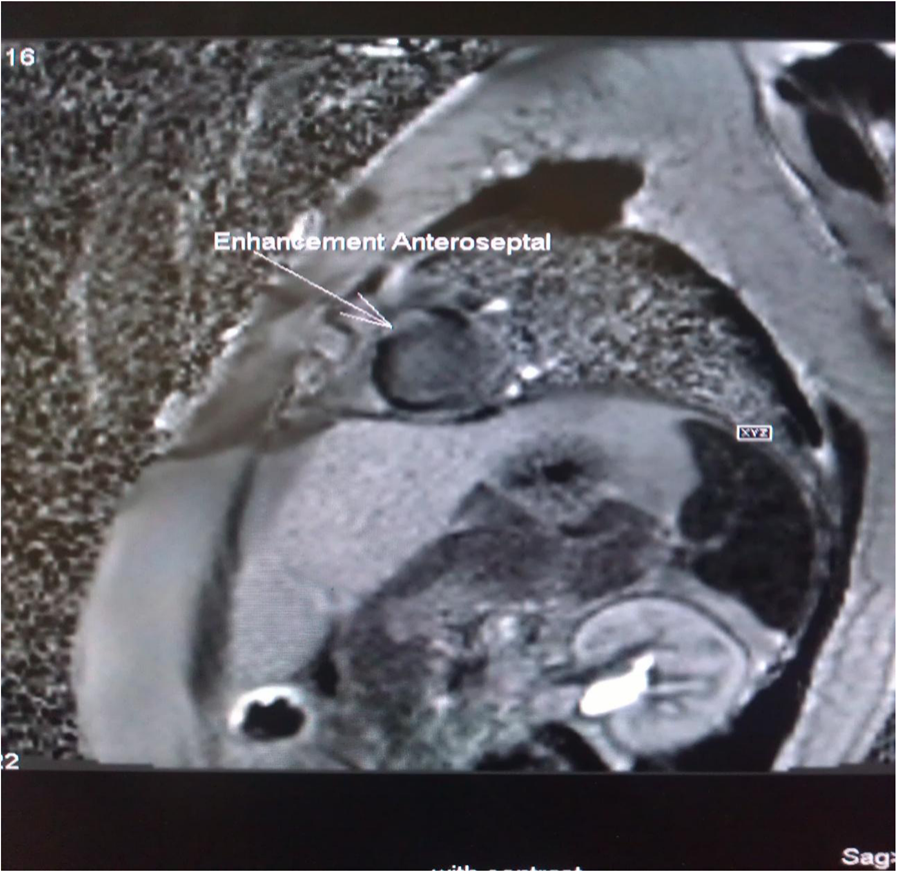
Cardiac magnetic resonance imaging (contrast enhanced) showing enhancement of anteroseptal left ventricular region (long arrow).

**Figure 6:**
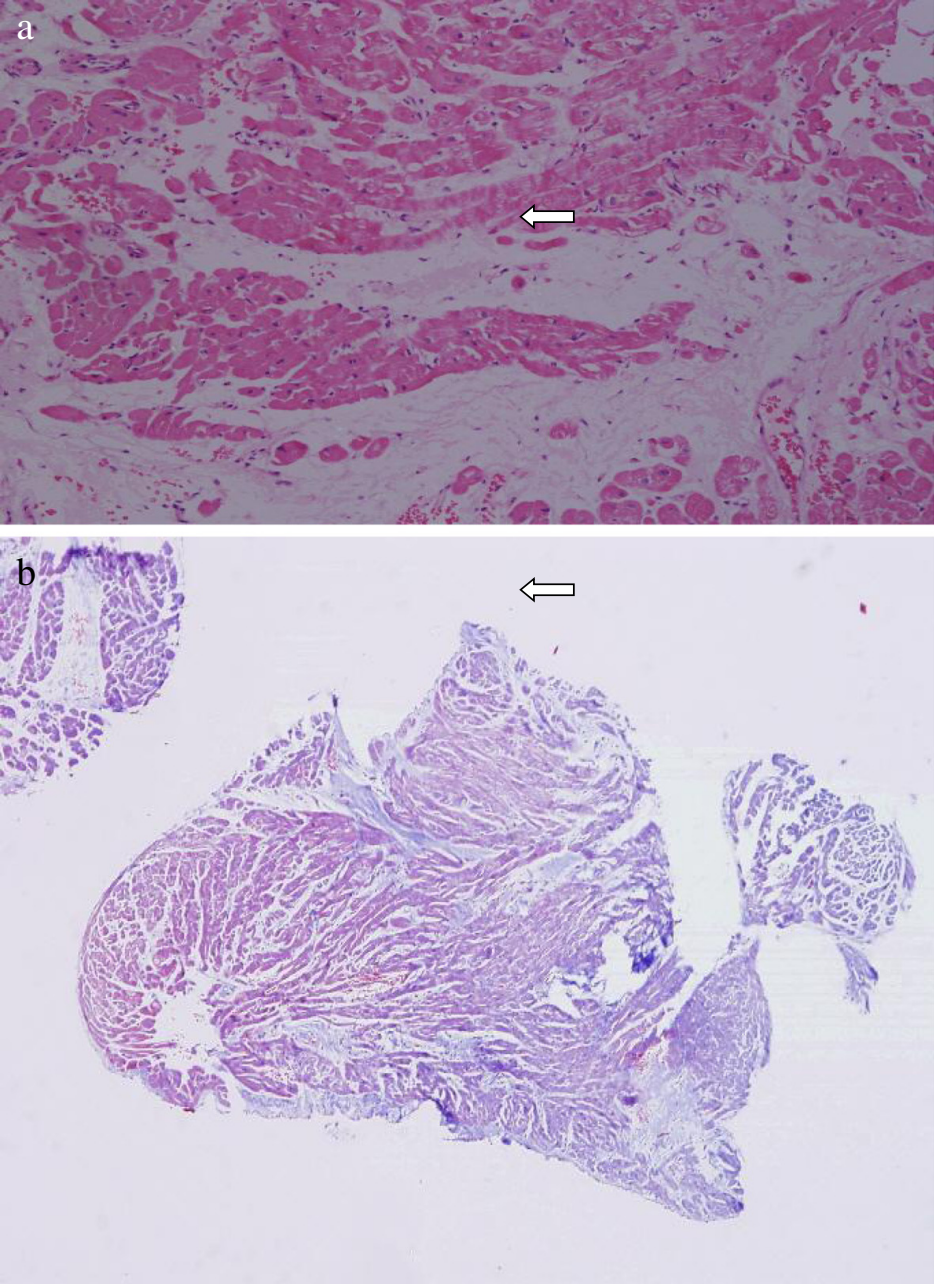
**Endomyocardial biopsies showing mild interstitial fibrosis (arrows). a**: ×100, hematoxylin and eosin stain. **b**: ×40, Masson trichrome stain.

Holter monitoring showed recurrent episodes of sustained monomorphic VT. Her coronary angiogram was normal. Her echocardiography revealed a left ventricular ejection fraction (EF) of 48% with hypokinesia of the mid-basal interventricular septum (IVS). Cardiac magnetic resonance imaging (MRI) with gadolinium contrast showed evidence of late enhancement in both the apical and anteroseptal left ventricular regions suspicious for fibrosis (Figure 5). To further confirm the diagnosis of cardiac involvement, endomyocardial biopsies were performed. However, her endomyocardial biopsies did not reveal granulomas, but showed focal interstitial fibrosis (Figure 6a, 6b).

On the basis of the above clinical and investigational findings, diagnosis of systemic sarcoidosis with cardiac involvement was made. She satisfied the Japanese Ministry of Health and Welfare guidelines for diagnosis of cardiac sarcoidosis (see section Guidelines for the diagnosis of cardiac sarcoidosis) [5]. She was started on prednisolone at a dose of 1mg/kg/day in addition to supportive care. She received multiple antiarrhythmic drugs including oral amiodarone at a maintenance dose of 300mg daily and metoprolol at a dose of 100mg twice daily without any satisfactory response. Radiofrequency ablation was attempted twice but failed to suppress the ventricular arrhythmia. She was then planned for automatic implantable cardiac defibrillator (AICD) implantation which could not be done due to financial constraints. In the following week, the patient died following an episode of VT that was unresponsive to defibrillation. The patient’s family refused autopsy studies.

### Guidelines for the diagnosis of cardiac sarcoidosis [5]

#### Histological diagnosis group

Cardiac sarcoidosis is confirmed when endomyocardial biopsy specimens demonstrate non-caseating epithelioid cell granulomas with histological or clinical diagnosis of extracardiac sarcoidosis.

#### Clinical diagnosis group

If endomyocardial biopsy specimens do not demonstrate non-caseating epithelioid granulomas, then extracardiac sarcoidosis is diagnosed histologically or clinically when the following combination of major or minor diagnostic criteria are satisfied:

1. Two or more of the four major criteria are satisfied

2. One in four of the major criteria and two or more of the five minor criteria are satisfied

#### Major criteria

1. Advanced atrioventricular block

2. Basal thinning of the interventricular septum

3. Positive gallium-67 uptake in the heart

4. Depressed ejection fraction of the left ventricle (<50%)

#### Minor criteria

1. Abnormal electrocardiogram findings: ventricular arrhythmias (ventricular tachycardia, multifocal or frequent premature ventricular contractions ), complete right bundle branch block, axis deviation or abnormal Q wave

2. Abnormal echocardiography: regional abnormal wall motion or morphological abnormality (ventricular aneurysm, wall thickening)

3. Nuclear medicine: perfusion defect detected by thallium-201 or technetium-99m myocardial scintigraphy

4. Gadolinium-enhanced contrast magnetic resonance imaging: delayed enhancement of myocardium

5. Endomyocardial biopsy: interstitial fibrosis or monocyte infiltration of moderate grade

## Discussion

Sarcoidosis is a multisystem granulomatous disease, which predominantly affects 20- to 30-year olds of both sexes. Most patients present with pulmonary involvement. In addition to the lungs, other affected organs are, in decreasing order of frequency, skin, liver, gastrointestinal tract, eyes and nervous system [6]. Cardiac involvement is one of the least common manifestations. In a recent review, the incidence of clinical heart involvement was reported as approximately 5%, whereas at autopsy the incidence was considerably higher (20 to 25%) [6-8].

The course of sarcoidosis can be indolent; however, acute complications in cardiac sarcoidosis can lead to sudden cardiac death [9,10]. Non-caseating granulomas serve as foci for abnormal automaticity and cause changes in the ventricular activation and recovery process, which explains the reentry mechanism that is thought to lead to VT in cardiac sarcoidosis [11].

Ante mortem diagnosis of cardiac sarcoidosis is difficult because of its wide-ranging clinical manifestations and the limitations of available diagnostic tests. Guidelines for the diagnosis of cardiac sarcoidosis have been published by the Japanese Ministry of Health and Welfare although they have not been validated (see section Guidelines for the diagnosis of cardiac sarcoidosis) [5,12]. Although possible cases of cardiac sarcoidosis should initially have an echocardiogram to look for supportive findings such as regional wall motion abnormalities, thickening of IVS with bright shadow consistent with infiltration and impaired left ventricular EF, these findings are not specific. Cardiac MRI with gadolinium enhancement and PET scanning are valuable aids in the diagnosis of myocardial sarcoidosis and are considered superior to gallium-labeled or technetium-labeled nuclear scans [13,14]. Reports suggest that the sensitivity of detecting sarcoid granuloma on endomyocardial biopsy is around 20%; hence a negative biopsy does not exclude the disease [15].

Sarcoidosis that involves the heart warrants prompt treatment with corticosteroids with or without other immunosuppressive agents [16]. A recent study showed that corticosteroids are more helpful in patients with mild to moderate left ventricular function impairment (left ventricular EF of 30 to 50%), whereas those with a severely reduced left ventricular EF of less than 30% in the late stage of disease did not benefit (probably because of irreversible myocardial damage and fibrosis) [17]. This is in keeping with our case where the patient presented in the late stages and did not show a response to steroids.

Ventricular arrhythmias are common in cardiac sarcoidosis but are often refractory to antiarrhythmic drugs including amiodarone. Most authorities recommend placement of an electronic pacemaker for complete heart block and an automatic implantable cardioverter-defibrillator for ventricular fibrillation or tachycardia and markedly reduced left ventricular EF [18]. Cardiac transplantation is a useful option in cardiac sarcoidosis refractory to medical management, however, some studies have shown a trend towards increased mortality [19,20]. However, with progress in prevention and treatment of ventricular arrhythmias, the primary cause of death in cardiac sarcoidosis has changed from sudden death to congestive heart failure.

## Conclusions

Cardiac involvement should be ruled out in any patients with systemic sarcoidosis. A negative endomyocardial biopsy should not be taken as evidence of the absence of cardiac sarcoidosis, particularly if suspicion is strong. Advances in cardiac imaging seem to be improving the diagnosis and, when highly suggestive, should prompt a search for a positive tissue biopsy in a visualized abnormal area of the myocardium or from a non-cardiac site. Steroid therapy seems to be beneficial when used relatively early in the course of the disease. Due to the risk of arrhythmia in this disease, a strong consideration for prophylactic implantation of an AICD should be considered, particularly in the presence of ventricular arrhythmias or reduced EF.

## Consent

Written informed consent was obtained from the patient’s relative for publication of this case report and accompanying images. A copy of the written consent is available for review by the Editor-in-Chief of this journal.

## Competing interests

The authors declare that they have no conflicting interests.

## Authors’ contributions

ME received the patient, collected data and wrote the paper. SS, RP, RN and NN were responsible for the patient, performed all the cardiac procedures, managed the patient and critically revised the manuscript. LS carried out the histopathological examination of tissue biopsies. All authors read and approved the final manuscript.
